# Neighborhood deprivation, breast cancer outcomes and stress-related gene expression in leukocytes and tumor tissue

**DOI:** 10.1186/s13058-025-02146-y

**Published:** 2025-11-04

**Authors:** Jie Shen, Yufan Guan, Joseph Boyle, Bernard F. Fuemmeler, Hua Zhao

**Affiliations:** 1 Department of Public Health Sciences, School of Medicine, University of Virginia, Charlottesville, Virginia 22903 USA; 2 Departments of Biostatistics, School of Public Health, Virginia Commonwealth University, Richmond, Virginia 23284 USA; 3 Departments of Family Medicine, School of Medicine, Virginia Commonwealth University, Richmond, Virginia 23284 USA

## Abstract

**Background:**

Neighborhood socioeconomic deprivation is a recognized contributor to disparities in breast cancer outcomes, yet the biological mechanisms linking neighborhood context to tumor behavior remain poorly defined. The conserved transcriptional response to adversity (CTRA), characterized by upregulation of pro-inflammatory genes and downregulation of type I interferon and antibody-related genes, may represent a stress-related immune dysregulation pathway relevant to cancer aggressiveness and survival.

**Methods:**

We examined associations among the Area Deprivation Index (ADI), CTRA gene expression profiles, tumor characteristics, and survival outcomes across two cohorts. **Cohort 1** included 467 breast cancer patients treated at M.D. Anderson Cancer Center, with RNA-seq data from peripheral leukocytes used to evaluate associations of ADI with CTRA and tumor characteristics at baseline. **Cohort 2** comprised 1082 TCGA breast cancer patients with RNA-seq data from tumor tissue analyzed to assess the prognostic relevance of CTRA. CTRA scores were calculated using established gene signatures. Multivariable regression and Cox proportional hazards models were applied.

**Results:**

In Cohort 1, higher ADI was significantly associated with elevated CTRA and pro-inflammatory gene expression in leukocytes (ρ = 0.082, *p* = 0.01). These immune profiles correlated with ER-negative status, stage III disease, and poor tumor differentiation. In TCGA (Cohort 2), elevated tumor CTRA expression was independently associated with worse overall survival (HR = 1.36, 95% CI: 1.04, 1.78, *p* = 0.02).

**Conclusions:**

These findings suggest that neighborhood socioeconomic disadvantage may influence systemic immune regulation through stress-related transcriptional responses, which in turn contribute to tumor aggressiveness and survival disparities in breast cancer.

**Supplementary Information:**

The online version contains supplementary material available at 10.1186/s13058-025-02146-y.

## Introduction

Neighborhood deprivation has been increasingly recognized as a contributor to cancer outcomes, particularly in breast cancer, where outcomes can vary significantly by race, socioeconomic status, and geography [1–5]. The Area Deprivation Index (ADI) is a well-validated metric that captures neighborhood-level socioeconomic disadvantage and has been linked to adverse cancer outcomes, including later-stage diagnosis, lower treatment adherence, and reduced survival [5–11]. However, the ways in which social adversity becomes “embedded” within biological systems that contribute to tumor progression and prognosis remain poorly understood.

One potential pathway connecting neighborhood deprivation to cancer biology is through chronic stress and its downstream effects on immune function and inflammation [12, 13]. The Conserved Transcriptional Response to Adversity (CTRA) is a well-characterized gene expression profile of immune function that includes upregulation of pro-inflammatory genes (e.g., *IL1β*, *IL6*), downregulation of type I interferon signaling (e.g., *IFI*, *OAS* family), and reduced antibody synthesis [14, 15]. Elevated CTRA has been observed in individuals experiencing chronic stress, low social support, and other adverse psychosocial conditions [14–19]. In the context of cancer, CTRA-related immune dysregulation may shape both the systemic immune environment and the tumor immune microenvironment, potentially accelerating tumor progression and weakening anti-tumor immunity [14, 20, 21].

Despite growing interest in stress-related transcriptional profiles, few studies have examined the relationship between neighborhood deprivation and CTRA gene expression in breast cancer patients. Moreover, it remains unclear whether CTRA activity is associated with clinically relevant tumor characteristics or patient survival, and whether such associations are evident in both peripheral leukocytes and bulky breast tumor tissue. To address these gaps, we evaluated the relationship between ADI and CTRA-related gene expression profiles in peripheral blood leukocytes from breast cancer patients (Cohort 1). We also assessed the association between the CTRA and aggressive tumor characteristics in Cohort 1. Then, we examined the prognostic significance of tumor CTRA gene expression using The Cancer Genome Atlas (TCGA) breast cancer dataset (Cohort 2). We posit that neighborhood disadvantage (ADI) is linked to a stress-related systemic immune milieu captured by leukocyte CTRA/pro-inflammatory expression, and that CTRA measured directly in tumor tissue indexes local inflammatory programs. These systemic and tumor signals are complementary, and each contributes to breast cancer aggressiveness and survival.

## Materials and methods

### Data sources

The study population for **Cohort 1**, included 467 breast cancer patients, derived from a breast cancer epidemiological study initiated in 2012 at The University of Texas MD Anderson Cancer Center (Houston, TX) [22]. Eligible participants were women with newly diagnosed, histologically confirmed stage I–III breast cancer, defined by the presence of malignant breast epithelial cells and classified by molecular subtype. All patients were residents of the Greater Houston area. Blood samples were collected prior to any cancer treatment, and race and ethnicity were determined through self-report. Written informed consent was obtained from all participants. Cohort 2 included 1082 breast cancer patients with available clinical, transcriptomic, and survival data from The Cancer Genome Atlas (TCGA) Program. The data for this study were downloaded directly from the NCI Genomic Data Commons (GDC) Data Portal (dbGaP Study Accession: *phs000178*; https://portal.gdc.cancer.gov/projects/TCGA-BRCA). The study protocol was approved by the Institutional Review Board at UVA.

### Area Deprivation Index (ADI)

In this study, we used the area deprivation index (ADI) from the Neighborhood Atlas to assess the levels of neighborhood deprivation [23]. The 2022 ADI was constructed using the 2014–2018 5-year estimates from the US Census American Community Survey. The description of Neighborhood Atlas and ADI can be seen at https://www.neighborhoodatlas.medicine.wisc.edu/. A neighborhood is defined as a Census block group. A census block is the smallest geographic unit used by the United States Census Bureau. A census block group comprises a set of blocks that collectively average 100 population and is the smallest unit with detailed demographic-economic characteristics. Thus, compared to traditional conceptualizations of a neighborhood, the Census block group is generally smaller, more homogeneous, and thus more informative. The ADI provides measures and rankings of neighborhoods by socioeconomic disadvantage in a region of interest. It includes factors for the theoretical domains of income, education, employment, and housing quality. This study used the national percentile rankings at the block group level from 1 to 100. The percentiles are constructed by ranking the ADI from low to high for all block groups in the US and grouping the block groups into bins corresponding to each 1% range of the ADI. Therefore, a block group with a ranking of 1 indicates the lowest level of disadvantage among US Census blogk groups, and an ADI with a ranking of 100 indicates the highest level of disadvantage. To confirm our results derived from the national percentile rankings, we also examined state-level percentile rankings. Specifically, we used the State of Texas rankings for Cohort 1.

### CTRA expression profile

In Cohort 1, expression values for all 53 genes in the CTRA profile were extracted from an existing RNA-seq dataset generated from leukocyte samples of 467 breast cancer patients. The raw counts were normalized using variance-stabilizing transformation (VST), followed by z-scoring of individual genes. Three CTRA-related scores were computed: (1) a pro-inflammatory score, defined as the sum of normalized expression values for 19 pro-inflammatory genes; (2) a type I interferon/antibody synthesis score, defined as the sum of 31 interferon-related and 3 antibody-related genes; and (3) the classical CTRA score, calculated as the difference between the pro-inflammatory score and the interferon/antibody synthesis score (CTRA = pro-inflammatory −  antiviral/antibody score). For the TCGA breast cancer cohort (Cohort 2), RNA-seq data from tumor tissue samples of 1082 breast cancer patients were retrieved from the GDC Data Portal. Expression values for 49 genes in the CTRA profile were extracted. Gene expression of *IL-8*, *IFIT1L*, *IGJ*, and *IGLL3* was excluded from the analysis due to undetectable expression levels in most samples. To handle missing expression data, multiple imputation by chained equations (MICE) [24] was employed.

### Statistical analysis

Area Deprivation Index (ADI) and CTRA gene scores were available for all patients. In Cohort 1, Pearson Correlation analysis was used to assess the relationship between ADI and CTRA in leukocytes. Then, multiple linear regression was used to assess associations between ADI and the CTRA composite score, pro-inflammatory gene score, and type I interferon/antibody synthesis score in leukocytes. Covariates included age at diagnosis, race, education, marital status, body mass index (BMI), physical activity, alcohol consumption, tobacco use, and estrogen receptor (ER) status (Table 2). Stratified analysis was performed to assess whether the correlation between ADI and the CTRA-related score in leukocytes differed by race/ethnicity, BMI category, and ER status. Then, we used logistic regression to estimate odds ratios (ORs) and 95% confidence intervals (CIs) for associations between CTRA-related scores in leukocytes (as exposure variables) and tumor characteristics (as outcome variables), including ER status, tumor stage, and histologic grade, adjusting for age at diagnosis and race (Table 3). For the TCGA breast cancer dataset (Cohort 2), Cox proportional hazards regression models were fit to evaluate associations between CTRA scores in breast tumor tissue and overall survival (Table 4). Covariates included age at diagnosis, self-reported race/ethnicity, menopausal status, AJCC tumor stage, tumor subtypes, tumor microenvironment characteristics, and cancer treatments. The tumor microenvironment was quantified using the xCell algorithm [25], which infers the relative abundance of immune and stromal cell types from bulk RNA-seq data using gene signature enrichment scores.

## Results

As Table 1 summarizes the epidemiological and clinical characteristics of breast cancer patients in Cohorts 1 and 2. The mean age at diagnosis was similar across cohorts (58.0 vs. 58.2 years). Most patients were White (69.6% in Cohort 1, 75.5% in Cohort 2), followed by Black (19.1 vs. 18.2%); In Cohort 1, 11.4% of patients were Hispanic, while in Cohort 2, 6.3% were Asian or Native American. In Cohort 1, the mean ADI was 56.4 (range: 5–80), nearly half of participants were married or living with a partner, 42% had less than a college education, and lifestyle factors reflected high prevalence of overweight/obesity (80.7%), former smoking (37.3%), and low physical activity (53.7%). With respect to tumor features, ER-positive disease predominated in both cohorts (70.5 vs. 77.0%), although Cohort 1 had a higher proportion of stage III tumors (35.1 vs. 23.7%) and poorly differentiated grade (48.4%). Treatment information available for Cohort 2 indicated that roughly half received chemotherapy, radiation, or hormone therapy.

**Table 1:** Breast cancer patient epidemiological and clinical characteristics in cohorts 1 and 2

	Breast cancer patient cohort 1	Breast cancer patient cohort 2
Variable	Breast cancer cases (n = 467), n (%)	Breast cancer cases (n = 1082), n (%)
ADI, mean (range)	56.42 (5, 80)	–
Age at diagnosis, mean (SD)	58.03 (14.33)	58.23 (7.6)
Race/Ethnicity		
White	325 (69.59)	746 (75.51)
Black	89 (19.06)	180 (18.22)
Hispanic	53 (11.35)	–
Asian/Native American	–	62 (6.28)
Marital status		
Married or living together	230 (49.25)	–
Single/Never married	138 (29.55)	–
Separated/Divorced/Widowed	99 (21.20)	–
Education		
College graduate/and above	117 (25.05)	–
Technical school/Some college	154 (32.98)	–
Below college	196 (41.97)	–
BMI		
Normal weight	90 (19.27)	–
Overweight	261 (55.89)	–
Obese	116 (24.84)	–
Smoking status		
Never	237 (50.75)	–
Former	174 (37.26)	–
Current	56 (11.99)	–
Alcohol drinking		
Never	125 (26.77)	–
Former	225 (48.18)	–
Current	117 (25.05)	–
Physical activity		
High	91 (19.49)	–
Medium	130 (27.84)	–
Low	247 (53.68)	–
ER status		
ER +	329 (70.45)	795 (77.03)
ER −	138 (29.55)	237 (22.97)
Tumor stage		
I/II	303 (64.88)	793 (76.33)
III	164 (35.12)	246 (23.68)
Tumor grade		
Well differentiated	98 (20.99)	–
Moderately differentiated	143 (30.62)	–
Poorly differentiated	226 (48.39)	–
Chemotherapy		
No	–	509 (47.04)
Yes	–	573 (52.96)
Radiation therapy		
No	–	502 (46.40)
Yes	–	580 (53.60)
Hormone therapy		
No	–	568 (52.50)
Yes	–	514 (47.50)

**Fig. 1:**
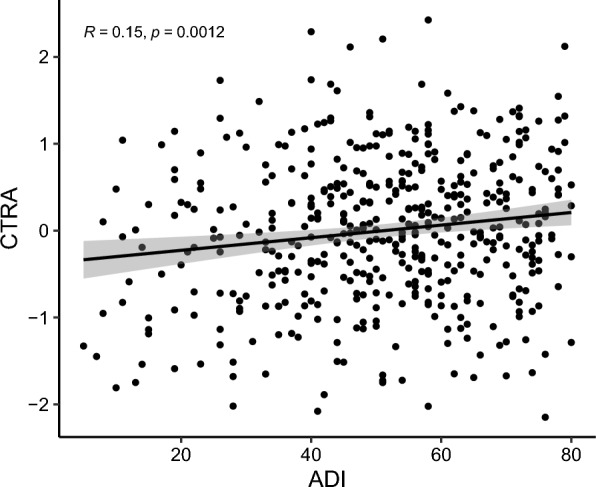
Correlation between ADI and leukocyte CTRA in breast cancer patients from Cohort 1

In Cohort 1, higher ADI was significantly and positively associated with elevated CTRA gene expression in leukocytes (ρ = 0.15, *p* < 0.01; Fig. 1). After adjusting relevant covariables, the significant association between ADI and CTRA gene expression in leukocytes remained (Table 2). In the overall sample, higher ADI was significantly associated with higher CTRA (β = 0.082, *p* = 0.01) and pro-inflammatory gene expression (β = 0.051, *p* = 0.01), and with lower type I interferon/antibody-related expression (β = −0.034, *p* = 0.04). Stratified analyses showed this pattern in both White and Black patients. Among White patients, ADI was positively associated with CTRA (β = 0.078, *p* = 0.03) and pro-inflammatory gene expression (β = 0.049, *p* = 0.04), with a non-significant inverse association for interferon/antibody expression (β = –0.029, *p* = 0.26). Among Black patients, associations were stronger, with significant positive associations with CTRA (β = 0.108, *p* = 0.03) and pro-inflammatory expression (β = 0.078, *p* = 0.03), and a negative, though non-significant, association with interferon/antibody expression (β = –0.037, *p* = 0.32). For Hispanic patients, ADI was positively associated with CTRA (β = 0.077, *p* = 0.09) and pro-inflammatory genes (β = 0.045, *p* = 0.10) and inversely associated with interferon/antibody expression (β = –0.033, *p* = 0.45), though none reached statistical significance. When stratified by BMI, associations were weakest among normal-weight patients (CTRA: β = 0.066, *p* = 0.24; pro-inflammatory: β = 0.035, *p* = 0.36), but stronger among overweight (CTRA: β = 0.085, *p* = 0.03; pro-inflammatory: β = 0.057, *p* = 0.02) and obese patients (CTRA: β = 0.094, *p* < 0.01; pro-inflammatory: β = 0.065, *p* < 0.01). In both overweight and obese groups, ADI was negatively, but non-significantly, associated with interferon/antibody expression (β = –0.035 and –0.036, respectively). Stratification by ER status revealed consistent associations in both subgroups: among ER-positive patients, ADI was associated with higher CTRA (β = 0.080, *p* = 0.04) and pro-inflammatory expression (β = 0.049, *p* = 0.04), with a negative but non-significant interferon/antibody association (β = –0.033, *p* = 0.22). Among ER-negative patients, associations were slightly stronger (CTRA: β = 0.086, *p* = 0.01; pro-inflammatory: β = 0.055, *p* = 0.01), with a consistent but non-significant negative association for interferon/antibody expression (β = –0.035, *p* = 0.29). In addition to national percentile rankings, we also used state-level percentile rankings from Texas for ADI. Consistent and significant associations were observed between ADI and both CTRA and pro-inflammatory gene expression scores in leukocytes.

**Table 2:** Relationships between ADI with CTRA and its component gene expression scores in leukocytes among breast cancer patients in Cohort 1

	CTRA	*P* value^*^	Proinflammatory	*P* value^*^	Type I interferon responses and antibody synthesis	*P* value^*^
All	0.082 (0.009)	**0.01**	0.051 (0.012)	**0.01**	−0.034 (0.011)	0.04
By race/Ethnicity						
White	0.078 (0.010)	**0.03**	0.049 (0.011)	**0.04**	−0.029 (0.012)	0.26
Black	0.108 (0.016)	**0.03**	0.078 (0.017)	**0.03**	−0.037 (0.016)	0.32
Hispanics	0.077 (0.013)	0.09	0.045 (0.020)	0.10	−0.033 (0.019)	0.45
By BMI						
Normal weight	0.066 (0.022)	0.24	0.035 (0.022)	0.36	−0.031 (0.022)	0.41
Overweight	0.085 (0.017)	**0.03**	0.057 (0.016)	**0.02**	−0.035 (0.015)	0.22
Obese	0.094 (0.019)	** < 0.01**	0.065 (0.018)	** < 0.01**	−0.036 (0.018)	0.15
By ER status						
Positive	0.080 (0.015)	**0.04**	0.049 (0.014)	**0.04**	−0.033 (0.014)	0.22
Negative	0.086 (0.019)	**0.01**	0.055 (0.018)	**0.01**	−0.035 (0.020)	0.29

^*^Adjusted by age at diagnosis, race, education, marital status, BMI, physical activity, alcohol drinking, tobacco use, and ER status as appropriate. Bold indicated P value <0.05

As Table 3 presents the associations between CTRA and its component gene expression scores in leukocytes with tumor characteristics among breast cancer patients in Cohort 1. For ER status, higher pro-inflammatory gene expression was associated with increased odds of ER-negative tumors (OR = 1.13, 95% CI: 1.04–1.50), whereas overall CTRA (OR = 1.06, 95% CI: 0.93–1.37) and interferon/antibody-related expression (OR = 0.71, 95% CI: 0.43–1.14) were not significantly associated. For tumor stage, elevated CTRA was associated with higher odds of stage III disease compared with stage I/II (OR = 1.12, 95% CI: 1.03–1.44). In contrast, pro-inflammatory gene expression (OR = 1.06, 95% CI: 0.80–1.61) was not statistically significant, while interferon/antibody expression was inversely associated with stage III disease (OR = 0.64, 95% CI: 0.32–0.97). For tumor grade, patients with poorly differentiated tumors had higher odds of elevated CTRA (OR = 1.14, 95% CI: 1.05–1.62) and pro-inflammatory gene expression (OR = 1.15, 95% CI: 1.05–1.69) compared with those with well- or moderately differentiated tumors. Interferon/antibody-related expression was inversely associated with poor differentiation (OR = 0.85, 95% CI: 0.60–1.22), though this was not statistically significant.

**Table 3:** Associations between CTRA and its component gene expression scores in leukocytes with tumor characteristics among breast cancer patients in Cohort 1

	CTRA	Proinflammatory	Type I interferon and antibody synthesis
	OR (95% CI)^*^	OR (95% CI)^*^	OR (95% CI)^*^
ER status: negative vs positive			
Positive	Reference	Reference	Reference
Negative	1.06 (0.93, 1.37)	**1.13 (1.04, 1.50)**	0.71 (0.43, 1.1.14)
Stage: III vs I/II			
I/II	Reference	Reference	Reference
III	**1.12 (1.03, 1.44)**	1.06 (0.80, 1.61)	**0.64 (0.32, 0.97)**
Grade			
well or moderate differentiated	Reference	Reference	Reference
Poorly differentiated	**1.14 (1.05, 1.62)**	**1.15 (1.05, 1.69)**	0.85 (0.60, 1.22)

^*^Adjusted by age at diagnosis and race. Bold indicated the association was statistically significant

Then, in Cohort 2, we examined whether CTRA and its component gene expression scores in breast tumor tissue differed by demographic and clinical characteristics (Supplement Table 1). Gene expression profiles did not differ significantly by age (all *p* > 0.18). Similarly, no significant differences were observed by race for pro-inflammatory (*p* = 0.62) or interferon/antibody-related (*p* = 0.15) scores. A borderline association was noted for overall CTRA (*p* = 0.06), with Asian/Native patients exhibiting slightly lower CTRA values compared with White and Black patients. Tumor stage was not associated with pro-inflammatory (*p* = 0.51) or overall CTRA (*p* = 0.12) scores; however, type I interferon/antibody-related expression differed significantly by stage (*p* = 0.02), with higher levels observed in stage II compared with stage I or III disease. No significant differences were observed across molecular subtypes for any of the gene expression measures (all *p* > 0.27).

As Table 4 shows the associations between CTRA and its component gene expression scores in breast tumor tissue with overall survival in Cohort 2. In crude analyses, higher tumor CTRA expression was associated with worse overall survival (HR = 1.26, 95% CI: 1.01–1.56, *p* = 0.04). This association was strengthened after adjustment for demographic and clinical covariates (Model 1: HR = 1.40, 95% CI: 1.07–1.83, *p* = 0.01) and remained statistically significant with additional adjustment for treatment factors (Model 2: HR = 1.36, 95% CI: 1.04–1.78, *p* = 0.02). Pro-inflammatory gene expression was not significantly associated with survival across models (Model 2: HR = 1.15, 95% CI: 0.74–1.78, *p* = 0.54). In contrast, higher interferon/antibody-related gene expression showed a trend toward better survival. While not significant in crude analyses (HR = 0.86, 95% CI: 0.68–1.07, *p* = 0.17), the association approached significance in Model 1 (HR = 0.78, 95% CI: 0.60–1.02, *p* = 0.07) and reached borderline significance in the fully adjusted model (HR = 0.76, 95% CI: 0.58–1.00, *p* = 0.05).

**Table 4:** Associations between CTRA and its component gene expression scores in breast tumor tissue with overall survival in the TCGA breast cancer cohort

	Model 1		Model 2	
	HR (95% CI)	*P* value	HR (95% CI)	*P* value
CTRA	**1.40(1.07,1.83)**	**0.01**	**1.36(1.04,1.78)**	**0.02**
Pro-inflammatory	1.26(0.83, 1.92)	0.28	1.15(0.74, 1.78)	0.54
Type I interferon responses and antibody synthesis	0.78(0.60, 1.02)	0.07	0.76(0.58, 1.00)	0.05

Model 1: age, race, menopause status, stage, tumor subtypes, and microenvironment

Model 2: age, race, menopause status, stage, tumor subtypes, microenvironment, chemotherapy, radiation, and hormone therapy. Bold indicated the association was statistically significant

## Discussion

In this study, we examined associations between neighborhood disadvantage, CTRA gene expression profiles, breast tumor characteristics, and survival outcomes in two breast cancer datasets. We found that higher ADI, a measure of neighborhood-level deprivation, was significantly associated with elevated CTRA composite scores and pro-inflammatory gene expression in peripheral leukocytes (Cohort 1). These transcriptional profiles were in turn associated with more aggressive tumor features, including ER-negative status, higher stage, and poor differentiation. In the TCGA dataset (Cohort 2), elevated CTRA gene expression in breast tumor tissue was linked to worse overall survival, underscoring the biological and clinical significance of stress-related immune dysregulation in breast cancer.

In this study, higher neighborhood deprivation (ADI), was consistently associated with elevated leukocyte CTRA and pro-inflammatory gene expression and reduced interferon/antibody-related expression. These findings align with the canonical CTRA framework, suggesting that adverse social environments may contribute to systemic immune dysregulation characterized by heightened inflammatory signaling and diminished antiviral/humoral responses [4, 14–19]. The subgroup analyses provided further insight into population heterogeneity. Associations were evident in both White and Black patients, but effect sizes were stronger in Black patients, consistent with evidence that structural disadvantage disproportionately impacts systemic inflammatory biology in this group [26, 27]. Among Hispanic patients, the associations trended in the same direction but did not reach significance, likely due to smaller sample size. Stratification by BMI revealed that associations between ADI and CTRA were strongest in overweight and obese women but attenuated in normal-weight patients. This suggests that obesity-related inflammatory pathways may interact with social adversity to amplify systemic immune activation. Similarly, stratification by ER status demonstrated consistent associations in both ER + and ER– patients, with slightly stronger effects in ER– disease. Given the aggressive clinical course of ER– tumors, these findings raise the possibility that stress-related immune dysregulation may contribute to disparities in tumor aggressiveness. Similar evidence from Barnard et al. found that while composite CTRA scores in breast tumor tissue did not differ by ADI or early life trauma, ER-negative cases with high ADI had significantly higher expression of pro-inflammatory and antibody-related genes [28]. Notably, the associations with type I interferon and antibody-related gene expression were consistently negative but rarely reached statistical significance in stratified models, despite being significant in the overall cohort. This pattern may reflect smaller subgroup sample sizes but also suggests that the downregulation of antiviral and humoral pathways may be subtler and more context-dependent than the robust upregulation of pro-inflammatory genes.

In Cohort 1, leukocyte-based CTRA gene expression profiles were associated with indicators of tumor aggressiveness. Although overall CTRA expression was not significantly associated with ER status, higher pro-inflammatory gene expression was linked to greater odds of ER-negative disease, a subtype associated with more aggressive clinical behavior and poorer prognosis. Strikingly, higher overall CTRA expression was associated with increased odds of stage III disease compared with stage I/II, suggesting that systemic stress-related transcriptional activity may parallel more advanced tumor progression. Within CTRA components, interferon/antibody-related expression was significantly inversely associated with stage III disease, consistent with the hypothesis that preserved antiviral/humoral immunity may constrain tumor advancement. Finally, both CTRA and pro-inflammatory gene expression were significantly associated with poorly differentiated tumors relative to well or moderately differentiated tumors, reinforcing the role of systemic inflammatory signaling in more aggressive histopathologic phenotypes.

In the TCGA cohort, tumor-based expression of the CTRA gene signature was significantly associated with overall survival. Patients with higher tumor CTRA scores had poorer survival in crude analyses, and the association was strengthened after adjustment for demographic and clinical covariates and remained significant when treatment variables were included. These findings suggest that stress-related transcriptional activity within the tumor microenvironment, beyond systemic leukocyte profiles, may have direct implications for breast cancer prognosis. When examining CTRA components, pro-inflammatory gene expression alone was not significantly associated with survival, indicating that the prognostic value of the CTRA signature cannot be attributed solely to inflammation. In contrast, type I interferon and antibody-related gene expression showed an inverse relationship with mortality, consistent with the idea that preserved antiviral and humoral immune activity may confer a protective effect. Although this association did not reach significance in unadjusted models, it approached significance with multivariable adjustment and achieved borderline statistical significance in the fully adjusted model.

When assessing the relationship between CTRA and its component gene expression scores in breast tumor tissue with demographic and clinical characteristics, type I interferon/antibody-related expression varied significantly by tumor stage, with higher levels in stage II compared with stage I or III, suggesting a potential transient immune activation in intermediate-stage tumors. A borderline association between race and overall CTRA was also observed, with Asian/Native patients tending toward lower CTRA values relative to White and Black patients, although sample sizes in this group were small. No significant differences were detected by age or molecular subtype.

This study has several limitations. First, the cross-sectional design of the gene expression data precludes causal inference between neighborhood disadvantage, stress-related transcriptional activity, and tumor characteristics. Second, although we adjusted for key demographic, lifestyle, and clinical covariates, residual confounding from unmeasured factors cannot be excluded. Third, analyses using TCGA supported the prognostic relevance of the CTRA signature but could not evaluate neighborhood context because ADI measures are not available, representing a major limitation in linking social exposures with tumor biology. Finally, while the CTRA gene set is well-validated in blood-based studies, its application to tumor tissue may require refinement to capture tissue-specific immune and stromal dynamics.

Despite these limitations, our findings provide evidence that neighborhood deprivation may influence breast cancer outcomes through systemic stress-related immune dysregulation. Higher ADI was associated with elevated leukocyte CTRA profiles, marked by increased pro-inflammatory signaling and reduced antiviral/antibody responses. Complementing this, analysis of the TCGA cohort demonstrated that higher tumor CTRA expression was independently associated with poorer overall survival, underscoring the prognostic relevance of this transcriptional program. Together, these results support a potential inflammation–immune pathway linking adverse neighborhood environments to breast cancer aggressiveness and outcomes, while highlighting the need for larger, integrative studies to validate these observations and clarify underlying mechanisms.

## Supplementary Information

Additional file1 (DOCX 18 kb)

## Data Availability

All data generated or analyzed during this study are included in this published article and its supplementary information files.
