# Supplementary material for: Neighborhood deprivation, breast cancer outcomes and stress-related gene expression in leukocytes and tumor tissue
Source: Breast Cancer Res. 2025 Nov 4;27:196. doi: 10.1186/s13058-025-02146-y (PMC12584520; doi:10.1186/s13058-025-02146-y)
Supplement: Supplementary file 1 — Additional file1 (DOCX 18 kb) [file 13058_2025_2146_MOESM1_ESM.docx]

**Supplement Table 1.** the relationship between CTRA and its component gene expression scores in breast tumor tissue with selected variables Adjusted by Microenvironment Score

|  | CTRA | | Pro-inflammatory | | Type I interferon responses and antibody synthesis | |
| --- | --- | --- | --- | --- | --- | --- |
|  | Mean (SD) | *P* value | Mean (SD) | *P* value | Mean (SD) | *P* value |
| Age |  |  |  |  |  |  |
| <=58 | -0.45 (0.91) | 0.93 | 4.34 (0.63) | 0.18 | 4.78 (0.84) | 0.38 |
| >58 | -0.45 (0.85) |  | 4.29 (0.57) |  | 4.74 (0.78) |  |
| Race |  |  |  |  |  |  |
| White | -0.45 (0.87) | 0.06 | 4.32 (0.60) | 0.62 | 4.76 (0.82) | 0.15 |
| Black | -0.40 (0.88) |  | 4.31 (0.61) |  | 4.71 (0.80) |  |
| Asian/Native | -0.71 (1.05) |  | 4.23 (0.63) |  | 4.94 (0.91) |  |
| Stage |  |  |  |  |  |  |
| I | -0.37 (0.90) | 0.12 | 4.33 (0.63) | 0.51 | 4.70 (0.76) | 0.02 |
| II | -0.50 (0.90) |  | 4.32 (0.60) |  | 4.82 (0.84) |  |
| III | -0.39 (0.78) |  | 4.27 (0.59) |  | 4.66 (0.78) |  |
| Subtype |  |  |  |  |  |  |
| Luminal A | -0.46 (0.87) | 0.59 | 4.30 (0.59) | 0.27 | 4.76 (0.80) | 0.77 |
| Luminal B | -0.38 (0.92) |  | 4.39 (0.65) |  | 4.77 (0.81) |  |
| HER2-enriched | -0.51 (0.97) |  | 4.35 (0.63) |  | 4.86 (0.80) |  |
| Basal-like | -0.46 (0.91) |  | 4.30 (0.53) |  | 4.76 (0.78) |  |
